# Tunnel widening after ACL reconstruction with different fixation techniques: aperture fixation with biodegradable interference screws versus all-inside technique with suspensory cortical buttons. 5-year data from a prospective randomized trial

**DOI:** 10.1007/s00402-023-05001-x

**Published:** 2023-08-05

**Authors:** Martin Eichinger, Martin Ploner, Gerald Degenhart, Ansgar Rudisch, Vinzenz Smekal, René Attal, Raul Mayr

**Affiliations:** 1 Department of Orthopedics and Traumatology, a.ö. Bezirkskrankenhaus St. Johann in Tirol, Tirol, Austria; 2 Department of Orthopedics and Traumatology, Medical University of Innsbruck, Anichstrasse 35, 6020 Innsbruck, Austria; 3 Department of Radiology, Medical University of Innsbruck, Innsbruck, Austria; 4 AUVA Trauma Center, Klagenfurt, Austria; 5 Department of Orthopedics and Traumatology, Feldkirch Academic Hospital, Feldkirch, Austria

**Keywords:** All-inside, Anterior cruciate ligament, Button fixation, Screw fixation, Tunnel enlargement, Tunnel widening

## Abstract

**Background:**

The aim of the present study was to examine tunnel widening and clinical outcomes after anterior cruciate ligament reconstruction (ACLR) using two different fixation methods: aperture fixation with biodegradable interference screws versus all-inside ACLR with suspensory cortical buttons.

**Methods:**

Tunnel widening was assessed using volumetric and diameter measurements on magnetic resonance imaging (MRI) scans directly after surgery, as well as 6 months and 2 and 5 years postoperatively. Clinical outcomes were assessed after 5 years with instrumented tibial anteroposterior translation measurement (KT-1000), single-leg hop testing, and the IKDC, Lysholm, and Tegner activity scores.

**Results:**

At the final follow-up, the study population consisted of 21 patients, 12 of whom underwent screw fixation and 9 of whom had button fixation. 3 patients with all-inside ACLR had sustained early repeat ruptures within 6 months after surgery and had to be excluded from the further analysis. With screw fixation, the tibial tunnel volume changed significantly more over time compared to all-inside button fixation, with a larger initial increase at 6 months (from postoperative 2.9 ± 0.2 to 3.3 ± 0.2 cm^3^ at 6 months versus 1.7 ± 0.1 to 1.9 ± 0.2 cm^3^) and a greater final decrease over 2–5 years postoperatively (from 3.1 ± 0.2 to 1.9 ± 0.2 cm^3^ versus 1.8 ± 0.2 ± 0.1 to 1.3 ± 0.1 cm^3^) (*P* < 0.001). The femoral tunnel volume remained comparable between the two groups throughout the follow-up period, with an initial 1.6 ± 0.1 cm^3^ in both groups and 1.2 ± 0.1 vs. 1.3 ± 0.1 after 5 years in the screw and button groups, respectively (*P* ≥ 0.314). The maximum tibial and femoral tunnel diameters were significantly larger with screw fixation at all four time points. Tibial diameters measured 11.1 ± 0.2, 12.3 ± 0.3, 12.3 ± 0.4, and 11.2 ± 0.4 mm in the screw group versus 8.1 ± 0.3, 8.9 ± 0.3, 9.1 ± 0.4 and 8.2 ± 0.5 mm in the button group (*P* < 0.001). Femoral diameters measured 8.6 ± 0.2, 10.5 ± 0.4, 10.2 ± 0.3, and 8.9 ± 0.3 versus 7.3 ± 0.3, 8.4 ± 0.4, 8.4 ± 0.3, 7.5 ± 0.3, respectively (*P* ≤ 0.007). Four patients (33%) in the screw group exceeded a diameter of 12 mm on the tibial side after 5 years versus none in the button group (not significant, *P* = 0.104). Tibial anteroposterior translation measurement with KT-1000 after 5 years was 2.3 ± 2.4 mm in the screw group versus 3.2 ± 3.5 mm in the button group (not significant, *P* = 0.602). There were no significant differences between the groups in any of the other clinical outcomes.

**Conclusion:**

Tibial tunnels in ACLR with screw fixation were associated with a larger increase in tunnel volume within the first 2 years and a greater decrease up to 5 years after surgery, while femoral tunnel volumes did not differ significantly. On the tibial side, the need for staged revision ACLR may be greater after biodegradable interference screw fixation if repeat ruptures occur, especially within the first 2 years after primary ACLR. Concerns may remain regarding a higher graft failure rate with all-inside ACLR.

**Level of evidence:**

II.

**RCT consort:**

NCT01755819.

## Introduction

Anterior cruciate ligament (ACL) reconstruction (ACLR) has become one of the leading surgical interventions in the field of sports orthopedics and traumatology throughout the world [1, 2]. The large numbers of primary ACLR procedures are proportional to repeat ACL ruptures and the need for revision ACLR. Revision ACLR needs to be thoroughly planned in each individual case, and the location and size of the pre-existing bone tunnels play a major role in the surgical strategy.

Postoperative tunnel widening (TW) is a frequently reported phenomenon when soft-tissue grafts are used for ACLR [3–9]. Although it has been reported that TW does not affect the clinical outcome after ACLR, large tunnels can compromise graft fixation in revision ACLR or even make a two-stage procedure necessary [4, 10–13]. Nonanatomical tunnel placement has been identified as a factor leading to TW due to the resulting nonphysiological forces, but the etiology of TW in anatomically positioned tunnels has not yet been fully clarified [5, 14]. Both mechanical and biological factors have been identified that may contribute to TW [4–7, 15–17]. The expression of these factors may vary with the fixation methods used in ACLR. It has been reported in experimental animal studies that micromotions at the tendon–bone interface cause TW [16, 17]. Biological factors include the immune response, the reaction to foreign materials, the surface area for tendon–bone ingrowth, and influx of synovial fluid into the tunnel [5, 15].

When interference screws are used, the graft is compressed against the tunnel wall, allowing aperture fixation close to the joint. This may reduce graft–tunnel motion and influx of synovial fluid. Concerns regarding interference screw fixation include initial tunnel widening during insertion of the screw, damage to the graft, foreign-body reactions, and poor integration of the screw [18, 19]. Biodegradable materials such as biphasic calcium phosphate and poly(l-lactide-*co*-d,l-lactide) (PLDLA) have been developed to allow osseous integration of the interference screws, ideally after full ingrowth of the graft, thereby reducing postoperative TW.

When all-inside ACLR techniques are used, the tendon graft can be placed in a bone socket on both the femoral and tibial side, and fixation is achieved using adjustable-length loop cortical button devices. Early graft integration can be achieved through full bone–tunnel contact and the absence of foreign material except for the securing sutures [20, 21]. General concerns with the use of suspensory cortical button fixation include what are called “windshield wiper” or “bungee” effects, referring to micromotions at the tendon–bone interface, as well as influx of synovial fluid with subsequent inflammatory reactions [16, 22–26]. With regard to all-inside ACLR, laboratory studies have raised concerns about graft and button loop elongation, potentially resulting in greater graft micromotion and increased postoperative knee laxity [16, 22–26]. Clinical studies, on the other hand, have reported good functional outcomes [27–30].

There is still a paucity of prospective randomized trials reporting tunnel volume changes and clinical outcomes after all-inside ACLR using adjustable-length loop cortical buttons on the femoral and tibial side in comparison with ACLR with aperture fixation using biodegradable interference screws. The aim of the present study was, therefore, to compare the two techniques in relation to postoperative TW and clinical outcomes, with a follow-up interval of 5 years after ACLR. Two hypotheses were raised: firstly, that ACLR with biodegradable interference screw fixation would result in less postoperative TW in comparison with all-inside reconstruction using extracortical button fixation; and secondly, that the two techniques would lead to comparable clinical outcomes.

## Methods

The methods used in this study have been described previously [31].

### Patients

Over a 3-year period between January 2013 and February 2016, a total of 47 patients were enrolled in a prospective randomized study. On a randomized basis, the patients were assigned either to the ACLR technique, using aperture interference screw fixation on the femoral and tibial sides; or to all-inside ACLR, using adjustable-length loop cortical button fixation. Eligible patients were assigned to the treatment arms using block randomization. The inclusion criteria for patients aged from 18 to 45 years were as follows: (1) clinical and MRI diagnosis of unilateral ACL rupture; (2) 12-month period between ACL injury and reconstruction; (3) Tegner activity score ≥ 5; and (4) normal contralateral knee. Exclusion criteria comprised total collateral ligament rupture; a full-thickness cartilage lesion; and MRI or arthroscopic evidence of an unstable longitudinal meniscus tear that would require meniscus refixation and alterations in the postoperative rehabilitation protocol.

MRI scans of the knee were carried out within 3 days after surgery and at 6 months, 2 years, and 5 years postoperatively.

The study protocol was approved by the ethics committee of the Medical University of Innsbruck (ID: UN4820 316/4.22). The study was planned and conducted in accordance with the Consolidated Standards on Reporting Trials (CONSORT) guidelines (NCT01755819). All of the patients provided written informed consent prior to surgery.

### Surgical technique

All of the operations were performed by the same two senior surgeons specialized in knee surgery (R.A., V.S.).

#### Screw fixation

**Fig. 1:**
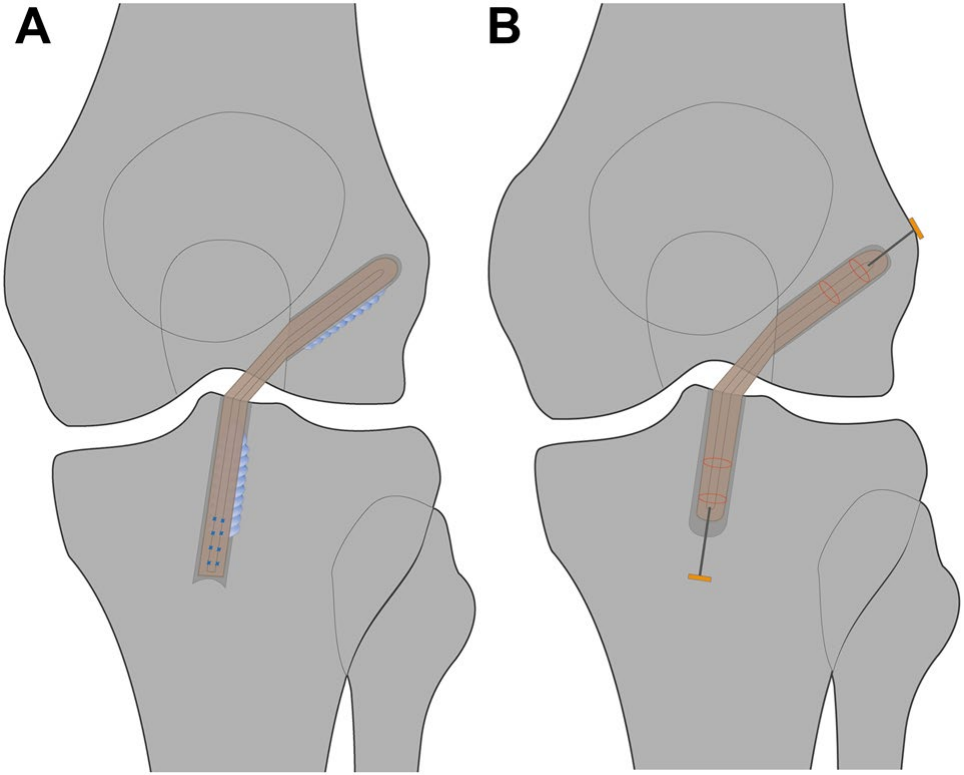
Anterior cruciate ligament reconstruction (ACLR) using (**a**) interference screw fixation with whipstitched tendon ends (blue dots) or (**b**) all-inside ACLR using button fixation with securing sutures (red lines)

The semitendinosus and gracilis tendons were harvested. The ends of the tendons were whipstitched using a nonresorbable suture (FiberWire #2; Arthrex Inc., Munich, Germany) (Fig. 1a). The tendons were folded to obtain a four-strand tendon graft, and the graft strands were sutured together with resorbable suture material on the femoral and tibial sides. On the femoral side, the mean size of the grafts was 7.3 ± 0.5 mm and on the tibial side it was 7.8 ± 0.8 mm. At 120° of knee flexion, the femoral tunnel was drilled through the anteromedial portal at the center of the femoral ACL insertion site to a length of 25 mm. A drill guide was used to create a full tibial tunnel in the tibial ACL stump, with the tibial ACL stump being preserved. The graft was pulled into the femoral socket. A bioabsorbable interference screw (BioComposite; Arthrex Inc.) 23 mm long, with a diameter 1 mm less than the femoral tunnel diameter, was inserted over a guide wire through the anteromedial portal. The knee was cycled approximately 10 times for graft preconditioning. The graft was fixed at the tibial site at 30° of flexion by inserting the bioabsorbable interference screw (BioComposite; Arthrex Inc.) using a guide wire. The screw length was 28 mm and the diameter was chosen 1 mm larger than the tibial tunnel diameter. The screw was inserted into the tibial tunnel aperture using the length scale on the screwdriver.

#### Button fixation

The semitendinosus tendon was harvested and folded over the loop of an adjustable-length loop cortical button (TightRope RT; Arthrex Inc.). The two ends of the tendon were whipstitched together using a nonresorbable suture (FiberWire #2; Arthrex Inc.) (Fig. 1b). The two tendon ends were passed through another cortical button loop to obtain a four-strand graft. The two ends of the graft were secured with two sutures (FiberWire #2; Arthrex Inc.) using the Lubowitz buried-knot technique [28]. The mean length of the tendon graft was 65.3 ± 4.9 mm. On the femoral side, the mean graft size was 7.8 ± 1.0 mm and on the tibial side it was 8.0 ± 0.6 mm. At the center of the femoral ACL insertion area, the femoral tunnel was drilled using the anteromedial (AM) portal reaming technique in two patients, or with an outside-in technique using a retrograde drilling guide pin in seven patients (FlipCutter; Arthrex Inc.). A retrograde drilling guide pin (FlipCutter; Arthrex Inc.) was used to create the tibial socket at the tibial ACL stump, preserving the stump as far as possible. A cortical bone bridge with a minimum of 7 mm was left. The graft was first pulled through the anteromedial portal into the femoral socket and then into the tibial socket. The knee was cycled approximately 10 times for graft preconditioning. Finally, the graft was tensioned by shortening the loop of the adjustable-length loop cortical buttons at the femoral and tibial sides at 30° of flexion.

### Rehabilitation

From the first postoperative day, the patients had active quadriceps exercise and passive knee motion, and full weight-bearing was immediately permitted. They wore knee braces for 2 weeks postoperatively. Cycling, muscle training, and swimming were allowed starting from weeks 4–12, and after 12 weeks running was permitted. Full exercise activity was allowed after 6–9 months.

### Clinical outcome

After a 5-year follow-up period, the clinical outcome was assessed using the International Knee Documentation Committee (IKDC) score, Lysholm score, Tegner activity score, hop testing, and KT-1000 measurement. The primary clinical outcome parameter was defined as anteroposterior stability in the knee after 5 years, assessed using the KT-1000 knee instrumented laxity measuring device (MEDmetric, San Diego, California).

### Imaging measurements

**Fig. 2:**
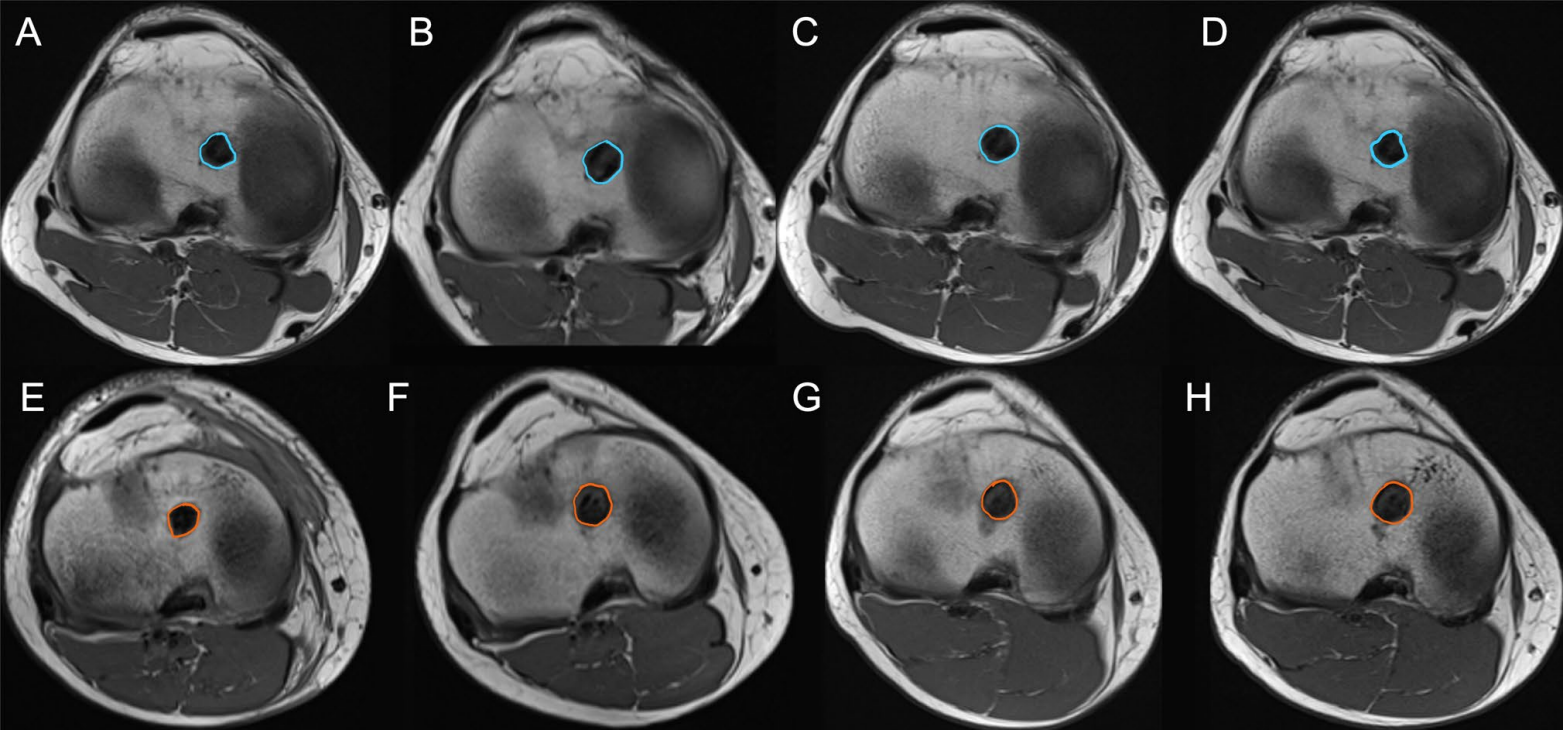
Tibial tunnels in one patient with interference screw fixation (blue, **a**–**d**) and one patient with button fixation (orange, **e**–**h**) at the four follow-up time points, on one representative axial slice each

Magnetic resonance imaging (MRI) was performed on the operated knee using a 1.5-T whole-body MR system (Magnetom Avanto; Siemens Healthcare Ltd., Erlangen, Germany) together with a 15-channel extremity coil. The bone tunnel volume was measured on axial sections of the turbo spin echo (TSE) T1 with a thickness of 3.0 mm. In the group with interference screw fixation, the screw volume was included in the measurement. The cross-sectional area of the bone tunnel was added together and multiplied to calculate the total volume on every slice (AW Server 2.0; GE Healthcare) (Fig. 2). An interrater intraclass correlation coefficient (ICC) of 0.656–0.920 has been reported with this measurement technique [32].

The MRI images, orientated along the longitudinal axes of the femoral and tibial tunnels, were used to measure the maximum diameters of the tunnel. ACL tunnel placement was measured on the sagittal slices of the postoperative MRI scan. The quadrant method on the lateral femoral condyle, as described by Bernard et al. [33, 34], was used to evaluate the femoral tunnel location. The center of the femoral tunnel was measured in the proximal–distal direction, normalized to the Blumensaat line, and the dorsal–anterior location was measured as the distance from the most posterior contour of the lateral femoral condyle. The interrater ICC has been reported to be between 0.729 and 0.895 [35]. The location of the tibial tunnel was evaluated along the distance from the anterior margin on the tibia to the center of the tibial tunnel (the Amis and Jakob line), expressed as a percentage of the anteroposterior tibial length [36], a measurement method for which an ICC of 0.934 has been reported [37].

### Statistical analysis

IBM SPSS Statistics, version 27.0 (IBM Corporation, Armonk, New York, USA), was used for statistical analysis. Parametric data are presented as means with standard deviation (SD). The groups were compared using the Mann–Whitney *U* test, regardless of normal distribution, to account for the small sample size. Categorical data were analyzed using Fisher’s exact test and expressed as absolute numbers and percentage distributions. Bonferroni correction was performed for repeated measures.

Changes in the absolute tunnel volume over time were compared between the two study groups using two-way mixed analysis of variance (ANOVA). To account for possible sphericity violation among states, the *P* values were corrected in accordance with the Greenhouse–Geisser method [38]. The *P* values reported are two-sided, and significance was set at < 0.05.

An effect size of 1.0 units was considered relevant for comparison of changes in tunnel widening between the two groups (difference in means: 10%, SD 10%), KT-1000 (2 mm, SD 2 mm), and the Lysholm score (2 points, SD 2). Achieving this with a power of 80% using a two-group comparison with a two-sided significance level of *P* < 0.05 requires a sample size of 17 in each treatment group. Data for the final follow-up were available for 12 patients with screw fixation and nine patients with button fixation, and 80% power was therefore not reached.

## Results

**Fig. 3:**
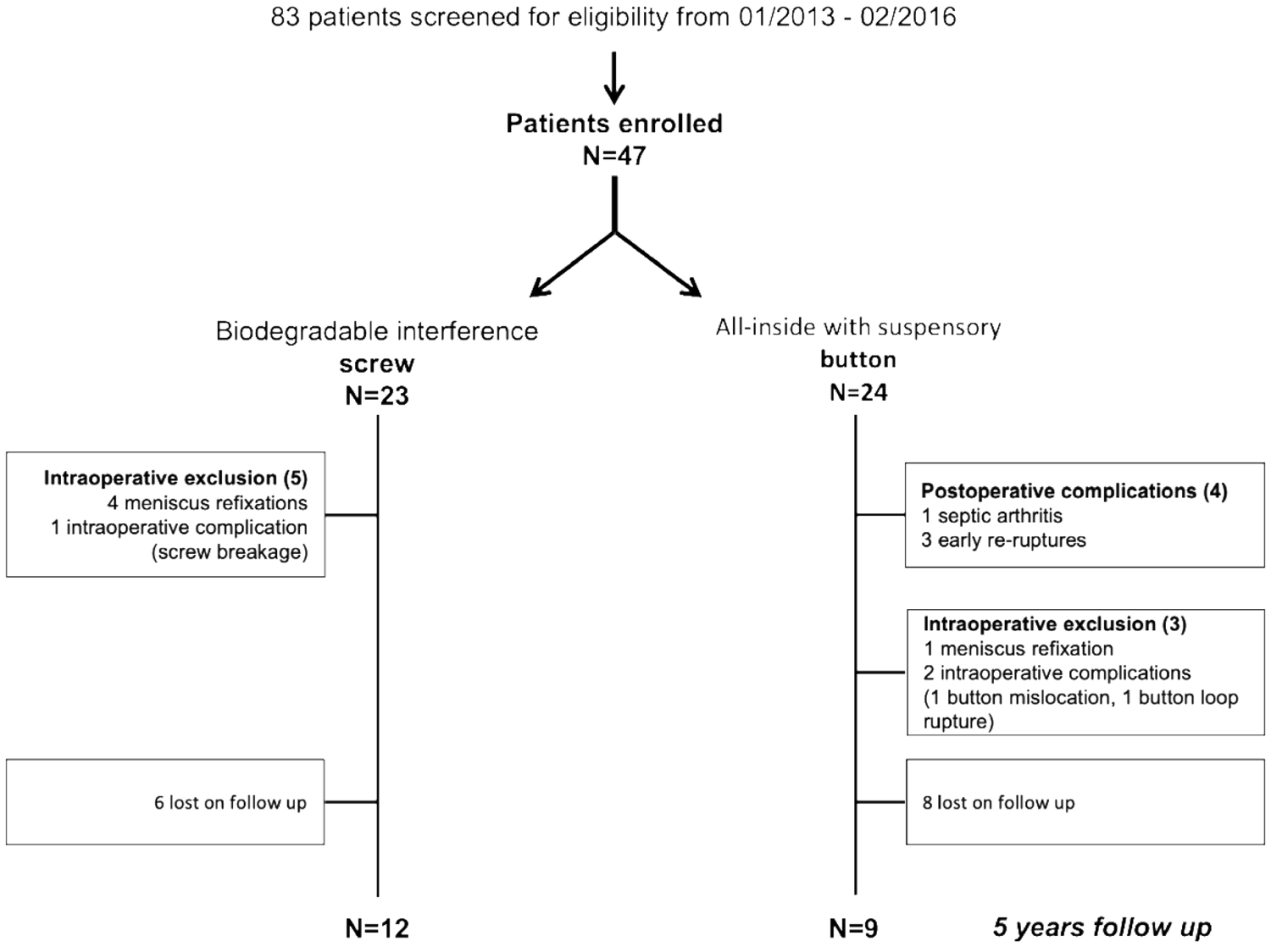
Patient demographics

The group with screw fixation had 23 patients allocated to it, and the all-inside reconstruction group with button fixation had 24 patients. Figure 3 shows the flowchart for the patients. Four patients with screw fixation and one patient with button fixation were excluded intraoperatively due to unstable meniscus tears. Intraoperative complications associated with the fixation technique included one femoral screw breakage, one button mislocation in the femoral tunnel, and one loop rupture of the femoral button. One patient with button fixation developed septic arthritis 2 weeks after the operation and received treatment with two irrigations and graft retention. ACL insufficiency was identified at the follow-up examination after 1 year, and the patient was excluded from the final analysis. One patient in the screw fixation group underwent a partial medial meniscus resection 1 year after ACLR and medial meniscal repair. Eighteen months after ACLR, one patient in the button fixation group underwent a repeat operation due to a cyclops lesion (localized anterior arthrofibrosis), with tibial button removal.

The patients’ demographic data showed no relevant differences in relation to age, sex, body mass index, or preinjury Tegner score (Table 1).

**Table 1:** Descriptive data of the study population

	Screw fixation (*n* = 12)	Button fixation (*n* = 9)
Age (y)	30 ± 7	27 ± 6
Sex (m, f)		
Female	4 (33%)	3 (33%)
Male	8 (67%)	6 (67%)
BMI	23.5 ± 2.0	23.3 ± 3.8
Tegner	7 (6–9)	7 (5–9)
Surgical time (min)	71.8 ± 23.2	87.6 ± 22.1
Partial meniscectomy (medial/lateral)	3 (2/1)	3 (3/0)
Meniscus refixation (medial/lateral)	1 (1/0)	0 (0/0)

Data are shown as means ± standard deviation, median (range), *n* (%). *BMI* body mass index

### Tunnel widening

**Fig. 4:**
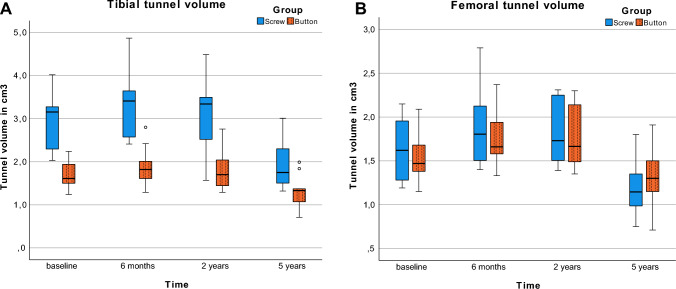
Tibial (**a**) and femoral (**b**) tunnel volumes after surgery and after 6 months, 2 years, and 5 years

In patients who underwent biodegradable interference screw fixation, the tibial tunnel volume (TV) in cm^3^ was significantly larger at all four measurement time points in comparison with all-inside suspensory cortical button fixation: 2.9 ± 0.2, 3.3 ± 0.2, 3.1 ± 0.2, and 1.9 ± 0.2 versus 1.7 ± 0.1, 1.9 ± 0.2, 1.8 ± 0.2 ± 0.1, and 1.3 ± 0.1, (*P* ≤ 0.009) (Table 2, Fig. 4).

**Table 2:** Tunnel volume and location

Group	Postoperative	Tibial tunnel volume (cm^3^)			Location (%)
		6 months	2 years	5 years	AJ
Screw	2.9 ± 0.2	3.3 ± 0.2	3.1 ± 0.2	1.9 ± 0.2	41.9 ± 8.2
Button	1.7 ± 0.1	1.9 ± 0.2	1.8 ± 0.2	1.3 ± 0.1	43.2 ± 2.7
*P*-value	< .001	< .001	.001	.009	n.s. (.666)

Group	Postoperative	Femoral tunnel volume (cm^3^)			Location (%)	
		6 months	2 years	5 years	PA	PD
Screw	1.6 ± 0.1	1.9 ± 0.1	1.8 ± 0.1	1.2 ± 0.1	24.5 ± 6.0	38.4 ± 8.8
Button	1.6 ± 0.1	1.8 ± 0.1	1.8 ± 0.1	1.3 ± 0.1	30.3 ± 6.6	36.6 ± 9.8
*P*-value	n.s. (.607)	n.s. (.520)	n.s. (.755)	n.s. (.314)	n.s. (.055)	n.s. (.666)

Data are shown as means with standard deviation

*AJ* tibial tunnel location along Amis and Jakob line in percent, *PA* posterior–anterior distance from posterior contour of lateral femoral condyle in percent, *PD* proximal–distal distance from Blumensaat line in percent

Tibial TV changes over time within 5 years after the index operation were significantly more dynamic in the biodegradable interference screw group in comparison with the group with all-inside suspensory cortical button fixation—i.e., there was a greater increase in the TV from baseline to the 6-month follow-up, developing to a greater decrease from the 2-year to the 5-year follow-up (*P* < 0.001) (Table 2, Fig. 4a).

The femoral TV in cm^3^ was comparable throughout all four measurement time points in the two groups and measured 1.6 ± 0.1, 1.9 ± 0.1, 1.8 ± 0.1, and 1.2 ± 0.1 in the screw group versus 1.6 ± 0.1, 1.8 ± 0.1, 1.8 ± 0.1, and 1.3 ± 0.1 in the button group, respectively, (*P* ≥ 0.314).The femoral TV changes over 5 years did not differ significantly (*P* = 0.080), (Table 2, Fig. 4b).

With regard to the comparison of the anteromedial technique and the outside-in femoral tunnel drilling technique in the group with button fixation no relevant differences were observed in femoral TV changes over time, (Appendix Table).

The maximum tibial and femoral tunnel diameters were significantly larger with screw fixation at all four time points. Tibial diameters in mm measured 11.1 ± 0.2, 12.3 ± 0.3, 12.3 ± 0.4, and 11.2 ± 0.4 in the screw group versus 8.1 ± 0.3, 8.9 ± 0.3, 9.1 ± 0.4 and 8.2 ± 0.5 in the button group (*P* < 0.001). Femoral diameters measured 8.6 ± 0.2, 10.5 ± 0.4, 10.2 ± 0.3, and 8.9 ± 0.3 versus 7.3 ± 0.3, 8.4 ± 0.4, 8.4 ± 0.3, 7.5 ± 0.3, respectively (*P* ≤ 0.007), (Table 3). After 5 years, six patients (50%) had a maximum tibial diameter of 10–12 mm and four (33%) had > 12 mm in the screw group, versus none for either 10–12 mm or > 12 mm in the button group (*P* = 0.002 and not significant with *P* = 0.104, respectively). On the femoral side, there were no patients with tunnels > 12 mm in maximum diameter. Two patients (17%) in the screw group had tunnels > 10 mm versus none in the button group (not significant, *P* = 0.486) (Table 3).

**Table 3:** Maximal tunnel diameter

Group	Postoperative	Diameter of the tibial tunnel (mm)			*N* (%) at 5 years	
		6 months	2 years	5 years	10–12 mm	> 12 mm
Screw	11.1 ± 0.2	12.3 ± 0.3	12.3 ± 0.4	11.2 ± 0.4	6 (50%)	4 (33%)
Button	8.1 ± 0.3	8.9 ± 0.3	9.1 ± 0.4	8.2 ± 0.5	0	0
*P*-value	< .001	< .001	.001	< .001	.002	n.s. (.104)

Group	Postoperative	Diameter of the femoral tunnel (mm)			*N* (%) at 5 years	
		6 months	2 years	5 years	10–12 mm	> 12 mm
Screw	8.6 ± 0.2	10.5 ± 0.4	10.2 ± 0.3	8.9 ± 0.3	2 (17%)	0
Button	7.3 ± 0.3	8.4 ± 0.4	8.4 ± 0.3	7.5 ± 0.3	0	0
*P*-value	.007	< .001	.001	.002	n.s. (.486)	

Data are shown as means with standard deviation

### Tunnel location

The tibial tunnel location was comparable between the two groups, with 41.9 ± 8.2 versus 43.2 ± 2.7% on the AJ line, (not significant, *P* = 0.666). A slightly more posterior femoral tunnel location was observed in the group with screw fixation (24.5 ± 6.0% in postero-anterior direction) in comparison with the group with button fixation (30.3 ± 6.6%), (not significant, *P* = 0.055) (Table 2).

### Clinical outcome

Of the initial 17 patients in the button group, 3 patients suffered early repeat ruptures within 6 months (one soccer injury, two distortions during everyday life) versus 0 out of 16 patients in the screw group (not significant, *P* = 0.227). One patient underwent single-stage revision ACLR with bone-patellar tendon-bone autograft, one patient underwent two-staged revision ACLR with quadriceps tendon autograft and one patient did not undergo revision ACLR at our institution.

At the final follow-up, three of 12 patients (25%) with screw fixation had KT laxity greater than 3 mm in comparison with four of nine patients (44.4%) with button fixation (not significant, *P* = 0.602). No significant differences were found in the IKDC objective and subjective scores (mean 92 ± 6 vs. 88 ± 17), Tegner activity score (mean 7; range 5–9 vs. 6; 4–8), or Lysholm scores (mean 90 ± 10 vs. 91 ± 11) at the final follow-up for screw and button group, respectively (*P* ≥ 0.247), (Table 4).

**Table 4:** Clinical outcome parameters

	Screw (*n* = 12)	Button (*n* = 9)	*P-*value
IKDC (No. of patients in A/B/C/D)			
Preoperative	0/0/6/3	0/0/9/0	n.s. (.345)
5 years	4/5/3/0	2/5/2/0	n.s. (.808)
IKDC subjective			
Preoperative	60 ± 8	65 ± 17	n.s. (.399)
5 years	92 ± 6	88 ± 17	n.s. (.437)
Pivot shift (0, glide + , clunk + + , gross + + +)			
Preoperative	2/9/1/0	2/5/2/0	n.s. (.808)
5 years	4/6/2/0	6/2/1/0	n.s. (.247)
KT-1000 side-to-side difference (mm)			
Preoperative	4.0 ± 1.8	6.6 ± 1.9	.004
5 years	2.3 ± 2.4	3.2 ± 3.5	n.s. (.602)
Lysholm			
Preoperative	73 ± 10	77 ± 15	n.s. (.602)
5 years	90 ± 10	91 ± 11	n.s. (.808)
Tegner			
Preoperative	7 (6–9)	7 (5–9)	n.s. (.808)
5 years	7 (5–9)	6 (4–8)	n.s. (.277)
Single leg hop (% of uninjured leg)			
Preoperative	68 ± 31	87 ± 13	n.s. (.310)
5 years	92 ± 26	94 ± 17	n.s. (.247)

Data are shown as means ± standard deviation, median (range)

*IKDC* International Knee Documentation Committee

## Discussion

The major finding of this study is that ACLR with biodegradable interference screw fixation is associated with significantly greater tibial tunnel widening in comparison with ACLR using extracortical button fixation. A greater increase in the tunnel volume was observed initially, with a stronger decrease after 2 years. The group with button fixation showed less tunnel widening on the tibial side, and the first hypothesis was therefore rejected. There were no significant differences in knee laxity and clinical outcome scores after 5 years, and the second hypothesis was thus accepted.

In the literature, several etiologic factors have been discussed that might contribute to tunnel widening after ACLR. These include simple displacement of an interference screw during insertion into the spongy bone of a tunnel already filled with a tendon transplant, inflammatory processes during conversion of biodegradable interference screws, local lysis effects during ingrowth of a tendon transplant, inflow of synovial fluid into the tunnels, and what are known as “bungee” or “windshield wiper” effects—i.e., micromotions of the tendons at the tendon–bone interface after suspensory cortical button fixation [4–7, 12, 14–17].

In the present study, there were greater tunnel volume changes over time after ACLR with aperture interference screw fixation on the tibial side, while on the femoral side the volumes were comparable throughout the follow-up.

The absolute values for the significantly larger postoperative tibial tunnel volume found in the present study need to be relativized by the fact that the transtibial tunnels needed for screw fixation are longer in comparison with the shorter socket used in the all-inside technique. Nonetheless, an initial tunnel widening effect through compression of the softer tibial spongy bone during insertion of an interference screw also needs to be taken into account, as reflected in the significantly larger maximum tibial diameters after screw fixation. These findings are consistent with those in a study by Monaco et al. [30], who reported more tibial tunnel widening after ACLR with biodegradable interference screw fixation on the tibial side and femoral suspensory cortical button fixation versus all-inside ACLR 1 year after surgery. The authors observed a mean tibial diameter increase of 2.42 ± 1.51 mm after screw fixation versus 0.81 ± 0.41 mm after all-inside ACL reconstruction, measured on CT scans. For the articular portion, values of 1.51 ± 0.81 mm versus 0.79 ± 0.78 mm were reported. Putnis et al. [39] recently reported similar results in a matched-cohort analysis that showed greater tibial TW with bioabsorbable interference screw fixation in comparison with tibial suspensory button fixation after 2 years. In a recent analysis, Liu et al. [40] reported significantly, eccentrically widened tibial and femoral tunnels 6 months after all-inside ACLR.

The tunnel diameter is an important factor during planning of ACLR in patients with a repeat ACL rupture. Staged revision needs to be considered if the tunnel diameter is greater than 10 mm and it may be indicated with diameters larger than 12 mm [10, 11].

In previously published findings for the same cohort of patients 2 years after the index operation, significantly more patients were found to have tibial tunnels wider than 10 or 12 mm after screw fixation in comparison with button fixation [31]. The present study shows that there are considerable changes in tibial TV over time after the use of interference screws, with a strong decrease from 2 to 5 years after surgery. At the final follow-up after 5 years, there were still significantly more patients with tunnels between 10 and 12 mm in diameter in the screw group in comparison with the button group. However, the difference was no longer significant for the benchmark of a tunnel diameter larger than 12 mm. Transferred to clinical practice, this might indicate a greater need for staged revision after tibial screw fixation in the case of repeat ACL rupture within 2 years after surgery, while this effect decreases after 2 years and beyond 5 years. On the femoral side, a comparable tunnel situation after screw or button fixation might be expected in the revision scenario. Reasons for the more dynamic change in tibial tunnel volume with biodegradable interference screw fixation observed in the present study might include: greater primary compression of the softer tibial spongy bone during insertion of an oversized interference screw; stronger local lysis effects at the tendon–bone interface, hypothetically due to stronger pressure at the interface; and inflammatory reactions within the first 2 years after ACLR, as well as biodegradation and osseointegration of the screws starting after 2 years and continuing to and maybe beyond 5 years, leading to reduction of the initially larger tunnel volumes.

On the femoral side, these effects seem less strong, and this might also be explained by the undersized interference screw and greater local bone density in comparison with the tibial tunnel.

The introduction of all-inside ACLR with adjustable-length loop cortical buttons on the femoral and tibial side provided an innovative technique without the need for full-length transosseous tunnels. As the technique came into widespread use, concerns were raised regarding potentially increased postoperative knee laxity and higher failure rates in comparison with conventional ACLR techniques [24–26, 31, 41, 42]. Some biomechanical studies have reported higher values for elongation of all-inside fixation versus aperture fixation [24–26], while others have not [43]. Similarly, there have been clinical studies reporting higher failure rates and/or postoperative knee laxity for all-inside ACLR versus aperture fixation [31, 41, 42], while others have reported comparable results [28–30]. To date, there is still no common consensus on the topic. A side-to-side difference of more than 3 mm in knee laxity is commonly regarded as failure of the ACLR [11, 44].

In the present study, the second hypothesis that there would be no differences in clinical outcomes between the study groups was accepted, as the differences in knee laxity as measured by the KT-1000 arthrometer were not statistically significant. However, this needs to be carefully discussed in the light of the study population and the current literature. Three patients in the group with all-inside ACLR with suspensory buttons sustained early repeat ruptures within 6 months after surgery versus zero in the screw group. Although, this was not significant, concerns on higher rerupture rates with the all-inside technique persist and must be further analyzed in future studies with higher patient numbers. Four of the remaining nine patients (44%) had knee laxity with more than 3 mm side-to-side difference after 5 years, resulting in a mean of 3.2 ± 3.5 mm, versus 2.3 ± 2.4 mm in the screw group. It should be taken into account that, despite patient randomization, the mean preoperative knee laxity was significantly greater in the button group than in the screw group, and this has been reported to be a risk factor for increased postoperative knee laxity [45]. Nonetheless, for comparison of clinical outcomes in failure rates and knee laxity, these figures argue in favor of ACLR with aperture fixation, despite the lack of statistical significance. Existing concerns regarding higher failure rates with all-inside ACLR cannot be relieved by these data. All other secondary clinical outcome parameters were comparable between the groups. Similar results have been published by Bressy et al. [41], who reported a high rate of postoperative side-to-side differences of more than 3 mm in 16 of 35 patients (46%) after all-inside ACLR with adjustable-length loop cortical buttons. A prospective randomized trial by Lubowitz et al. [29], a retrospective study by Monaco et al. [30], and a matched cohort analysis by Putnis et al. [39] comparing all-inside ACLR with ACLR using interference screw fixation all reported comparable knee laxity values and clinical outcome scores. These data have been summarized in three systematic reviews, which concluded that further studies with greater power and thorough follow-up designed for comparison of clinical outcomes between all-inside and conventional ACLR will be needed to clarify this question [42, 46, 47].

The main limitations of the present study include its power and the dropout rate. The high rate of dropouts was not expected, and the calculated sample size of 17 patients per group to achieve a power of 80% was not reached. The study must be regarded as underpowered, with a high potential of type I error on the primary outcome parameters. Since the study started only shortly after the introduction of the all-inside ACLR technique in our department, a learning curve with possible later improvements in the technical performance of a new technique over time must be regarded as a limitation of the study. Strengths of the present study are its prospective randomized design, with detailed follow-up including longitudinal tunnel volume assessment on MRI scans at four measurement time points within 5 years after surgery.

## Conclusion

Tibial tunnels in ACLR with biodegradable interference screw fixation were associated with a greater increase in tunnel volume within the first 2 years as well as a greater decrease up to 5 years after surgery, while femoral tunnel volumes were not significantly different. On the tibial side, the need for staged revision ACLR may be greater after biodegradable interference screw fixation in the case of repeat rupture, especially within the first 2 years after primary ACLR. Concerns may remain regarding a greater graft failure rate when all-inside ACLR is used.

## Data Availability

The datasets generated and analyzed during the current study are available from the corresponding author on reasonable request.

## Appendix

See Table

**Table 5:** Tunnel volume and location after femoral anteromedial or outside-in femoral tunnel drilling in all-inside ACLR

Group button	Postoperative	Femoral tunnel volume (cm^3^)			Location (%)	
		6 months	2 years	5 years	PA	PD
AM (*n* = 2)	1.7 ± 0.3	1.9 ± 0.3	2.0 ± 0.4	1.5 ± 0.1	30.8 ± 1.9	44.1 ± 0.9
OI (*n* = 7)	1.5 ± 0.3	1.7 ± 0.3	1.7 ± 0.4	1.3 ± 0.4	30.1 ± 7.5	34.5 ± 10.2

Data are shown as means with standard deviation

*AM* anteromedial drilling technique, *OI* outside-in drilling technique. *PA* posterior-anterior distance from posterior contour of lateral femoral condyle in percent, *PD* proximal–distal distance from Blumensaat line in percent

5.
